# Long‐Term Limiting Illness in the United Kingdom: Before and After the Covid Lockdown

**DOI:** 10.1002/hpm.3920

**Published:** 2025-03-05

**Authors:** Vani K. Borooah, Colin G. Knox

**Affiliations:** ^1^ Ulster University Belfast UK; ^2^ Nazarbayev University Astana Kazakhstan

**Keywords:** COVID, labour market, long‐term limiting illness

## Abstract

The purpose of this paper is to study the evolution of LTLI in the UK between the pre‐ and post‐Covid years of, respectively, 2019 and 2022 paying attention to differences in the propensity to LTLI between different subgroups of the population *in* each of the two years and then examining whether the propensity to LTLI changed *between* the years, both in respect of overall change and in respect of the separate population subgroups. This was achieved using UK Labour Force Survey data for 2019 and 2022. In terms of the social gradient to health, persons in the Managerial/Professional classes had a significantly *higher* PP (predicted probability) of N‐LTI (i.e., of not having a long‐term illness) than persons either in the Routine non‐Manual or Routine Manual classes and also had a significantly *lower* PP of LTLI‐lot (i.e., of having a long‐term illness which limited activity by a lot) than persons either in the Intermediate or in the Routine Manual or Routine non‐Manual classes. This was true in both 2019 and 2022. In other words, there was significant inequality in the PP of LTLI associated with the occupational classes. In terms of changes in the propensity to LTLI, the PP of having a long‐term illness—regardless of whether it was limiting or not ‐ was significantly *higher* in 2022 than it was in 2019 both for the overall population and for its subgroups.

**Summary**

- Existing health inequalities associated with LTLI have worsened since the pandemic.
- Those with LTLI are more likely to become economically inactive.
- Those with LTLI are more likely to have higher levels of sickness.
- LTLI has a significant impact on the economy.

## 1 Introduction

The publication of the Black Report, over 40 years ago, ushered in a new research theme centred around the social factors underlying illness and mortality [1]. This research has taken two routes. The first has been about mortality and the second has been about illness. With respect to mortality, the major conclusion of research can be summarised as: ‘wherever you stand on the social ladder, your risk of early death is higher than it is for your betters’ [2]. For example, Hattersley (1997) showed, after an analysis of longitudinal data for Britain, that there were stark inequalities in life expectancy by social class: the life expectancy of men and women in Social Class IV/V were, respectively, 70 and 77 years compared to the life expectancy of 75 and 80 years of men and women, respectively, in Social Class I/II [3]. Using a longer time frame, Marmot et. al. (2010) [4] found that although life expectancy had increased for everyone between 1971 and 2005, the gap in life expectancy by social class for both men and women persisted, with some widening taking place in the 1980s and 1990s. In the 10 years following the publication of the first Marmot Report (Marmot, 2010), Marmot et. al. (2020) [5] found that increases in life expectancy in England slowed since 2010 with the slowdown greatest in the more deprived areas of the country.

The second, and less‐travelled route, has been in respect of health inequalities in morbidity rates. For example, as Borooah (1999) noted [6], of the 11 chapters in Drever and Whitehead's (1997) [7] review of health inequalities in Britain, nine concerned mortality and just two were about morbidity. Nor has this trend to identify health inequality with inequality in life expectancy diminished over time. Marmot et. al. (2010) largely discussed health inequality in terms of differences in life expectancy between social groups and discussed in some detail the factors that might underpin such inequality: inequalities in the conditions of daily life—differences in the conditions in which people are born, grow, live, work and age—and the inequities in power, money and resources that might underpin these differences. The conflation of health with life expectancy did not alter when Marmot et. al. (2020) [5] conducted a retrospective of the original Marmot Report (i.e., Marmot et. al. 2010) [4].

This relative neglect of morbidity vis‐à‐vis mortality may be explained by the fact that morbidity embraces a wide array of ailments, and it may be difficult to arrive at summary measure of ill‐health with the same facility that measures of life expectancy provide (Bunting, 1997) [8]. Since 1991, however, a summary measure of a person's morbidity status is—and has been since the 1991 Census ‐ available for the UK (and its countries) in terms of whether a person has a *long‐term limiting illness (LTLI)*. By this is meant any long‐term illness, health problems, or handicap which limited one's daily activities, or the work one could do. As Dale and Marsh (1993) [9] observed, LTLI correlated well with the use of GP services and the use of in‐ and out‐patient use of hospital services.

This paper uses LTLI as a measure of morbidity status largely because of the term ‘limiting’ which, in turn, restricts activity and may, thereby, restrict one's capacity to work. As discussed below, the link between morbidity and employment is a matter of considerable policy concern in the UK today and the morbidity‐employment link is probably measured by using LTLI as a surrogate for morbidity. In a more general context, self‐rated health (SRH: where a person is asked to evaluate his/her health as: ‘excellent/very good’; ‘fair’; ‘poor’) is a commonly used measure of global morbidity. Several studies have also examined social inequalities and self‐rated health (Power et al, 1998 [10]; McFadden et al, 2008 [11]; Moor et al, 2017 [12]; Torsheim et al. 2018 [13]). Manor et. al. (2001) [14] have shown that these two measures, LTLI and SRH, were strongly associated with each other as well as with specific health problems, particularly those associated with serious conditions.

Lahbib et al. (2024) [15] used data from four French Health Barometer Surveys (2017, 2019, 2020, and 2021) to assess the evolution of SRH and activity limitation (measured by a Global Activity Limitation Indicator—GALI) of people in France aged 18–75 years. Their results are discussed in our concluding section in relation to the empirical evidence in our study of the UK. Suffice it to observe here, that the GALI is a single question instrument which asked respondents how limited, because of a health problem, they were, over the past 6 months, in activities people usually did with possible responses: not at all limited; limited but not severely; severely limited. By construction, GALI is very similar to the LTLI indicator used in UK Labour Force Surveys and both have the advantage over SRH that they explicitly incorporate the notion of ‘limiting’. However, all three measures suffer from the defect that they are self‐assessed.

This raises the question of whether, as self‐assessed measures, LTLI (and, its European counterpart, GALI) and SRH are good indicators of observed health. Cramm et al. (2015) [16] found only weak correlation between those ‘at risk’ on SRH and those at risk on objective indicators ‐ grip strength, lung capacity, and the degree of dependence in Activities of Daily Living (ADL) ‐ and suggested, as had been done earlier by Maddox and Douglass (1973) [17], that the two sets of measures ‘reflected different “parts” of mortality and morbidity’ (p. 256).

More recently, concern has been expressed about the effects of LTLI keeping people out of the labour force (i.e., either working or actively looking for work). The Office for National Statistics (ONS) reported that the number of ‘economically inactive’ working age adults (i.e., not in the labour force) citing LTLI as the primary reason for their inactivity, rose from around 2 million in the spring of 2019 to about 2.5 million by the summer of 2022 (ONS, 2022) [18]. Of people in employment, those with a LTLI had higher sickness absence rates (4.9%) than those without (1.5%). According to figures from the Office for National Statistics, economic inactivity was around three times higher among people with LTLI than those without (ONS, 2022 and 2023b) [18, 19]. In short, those with LTLI were more likely to be economically inactive or, if in work, to have higher rates of sickness. LTLI needs to be distinguished from long term sickness which is a period of continuous absence from work for 4 weeks or more and can be the result of an unexpected illness, a persistent condition (but not a LTLI) or an accident at work.

Against this background, there is a compelling case for studying LTLI with a view to answering two important questions *in the specific context of the Covid pandemic*. First, was the social gradient to health, by which the ‘rich’ live longer than the ‘poor’, mirrored in the context of LTLI both immediately before and after the pandemic? More specifically, after controlling for non‐class attributes, was there a social gradient to LTLI such that the probability of persons from the lower occupational classes (or from non‐white ethnicities) having a LTLI was greater than those from the higher classes (or persons whose ethnicity was white)? Second, comparing the year just before the Covid pandemic (2019) with that just after (2022), was the propensity to LTLI aggravated by the pandemic? Or, in other words, was there a significant difference between the pre‐ and post‐Covid periods in the gradient of the LTLI function with respect to the various population subgroups?

Since the thrust of this paper is with respect to inequalities in the propensity to LTLI with respect to ‘social position’ it is important to clarify our use of this term. Social position in this paper is identified with occupation: (i) **Managerial/Professional** (comprising, as listed in the LFS, Managers, Directors, and Senior Officials); (ii) **Intermediate** (comprising, as listed in the LFS, Administrative and Secretarial Occupations, Skilled Trades Occupations, and Caring, Leisure And Other Service Occupations; (iii) **Routine non‐Manual** (comprising, as listed in the LFS, Sales and Customer Service Occupations); and (iv) **Routine Manual** (comprising, as listed in the LFS, Process Plant, and Machine Operatives, and Elementary Occupations). It is important to emphasise that, on the face of it, these occupations categories are not hierarchical and so there may not be a direct link between them and higher and lower social positions.

With this clarification, it is important to emphasise that the measures of social class that we have used, based on occupational class, are not dissimilar to those used by others drawing on different definitions. For example, Bartley and Plewis (2002) [20], who carried out a longitudinal study of LTLI for the period 1971–91, used social class as defined by the Registrar General. This classification is described as ‘general standing in the community’ and is based on occupational skill. Class I, comprising professionals and managers of large businesses, is the ‘highest level’, and class V, unskilled manual workers, is the lowest level with a distinction in Class III being drawn ‐ as in this paper too ‐ between routine non‐manual jobs and the more skilled manual jobs.

An alternative to measuring social position by occupation is to use area deprivation as a measure of the same. Curtis (1990) [21] examined the relation between neighbourhood and health using a survey from three London health districts. The problem with this approach is that is needs small areas that are relatively homogenous in terms of socioeconomic factors. As the size of the area expands, the element of area heterogeneity increases with the result that there might be several rich/poor persons in poor/rich neighbourhoods. Given that the LFS areas were defined in terms of countries, this approach was not feasible for this study.

With this motivation for the paper, the data on which the analysis is based was drawn from the UK's Labour Force Survey (UK‐LFS) for 2019 and 2022 (October‐December for both years). The UK‐LFS asked its respondents whether they had a long‐term illness. If the answer was yes, they were asked if their long‐term illness *limited* their activity? If the long‐term illness was limiting (i.e., the respondent had a LTLI), the UK‐LFS then enquired as to whether the person's activity was limited ‘*a little*’ or ‘*a lot*’. So, the paper analyses a four‐part dependent variable *for each individual respondent of working age* (i.e., aged 16–64) such that:

- The respondent did not have a long‐term illness.
- The respondent had a long‐term illness, but it did not limit activity.
- The respondent had a long‐term illness, and it limited activity ‘a little’.
- The respondent had a long‐term illness, and it limited activity ‘a lot’.

There are various frameworks which classify long‐term illness [the World Health Organisation international classification of functioning, disability and health; the Global Burden of Disease study; surveys such as the UK Census and EU Statistics on Income and Living Conditions; and the Short Form health survey (SF‐36)]. Although classifications vary, some common distinctions include long‐term illness without limitation; long‐term illness with some limitation; and long‐term illness with severe limitation. The UK‐LFS broadly aligns with this classification and those used in the Equality Act 2010 which considers a person disabled if they have a physical or mental impairment that substantially limits their ability to perform normal daily activities.

Using a multinomial logit model, the paper then computes the probability of working‐age respondents to the UK‐LFS being in one of these four categories, first for *each* respondent and then, using these individual probabilities, the *average* for respondents in the different subgroups. Following this, the paper computes the likelihood of a person with a LTLI having various health conditions. Lastly, the paper compares the probabilities of persons having a LTLI between 2019 and 2022 and tests whether the pandemic significantly altered these probabilities both in aggregate and for the narrower categories of population subgroups and specific health conditions.

## 2 Prior Research on Long‐Term Limiting Illness

In broad terms existing scholarship on LTLI clusters around four thematic areas: the link between socio‐economic disadvantage and LTLI; the geographical variation in the prevalence of LTLI; the link between those presenting with LTLI and other illnesses; and the impact on individuals living with LTLI. We summarise findings from key works in these areas.

### 2.1 Disadvantage and LTLI

Early research using random sample data from the 1981 census (*n* = 300,000) investigated the association between the level of social deprivation in electoral wards and various life events including self‐reported long‐term limiting illnesses (Sloggett and Joshi, 1998) [22]. The researchers found a strong association between those living in more deprived areas and long‐term limiting illnesses. Living in deprived areas was associated with personal disadvantage which is more damaging to life chances. Deprivation indicators were therefore seen as good predictors of self‐reported long‐term limiting illnesses.

Borooah's study (1999) used 1991 census data to investigate the relationship between occupational class and health inequality. The study found that occupational class was important in explaining the probability of having a LTLI [6]. A subsequent paper (Borooah, 2007) [23] examined whether there was a social gradient to health in Northern Ireland (*n* = 2700) and found evidence that those who live in poor housing, without educational qualifications, and lower levels of income were more likely to suffer LTLI. Similar results (Archer et al., 2020) emanated from research which examined longitudinal associations between ‘socio‐demographic exposures’ and LTLI using data from the 2011 ONS Longitudinal Study of 2011 and the 2013 National Child Development Study [24].

Saul and Payne (1999) looked at differences between the health of social groups in Sheffield Health Authority (*n* = 16,751) to assess whether there were some indicators of socio‐economic disadvantage that were better than others at predicting the prevalence of specific morbidities at a population level [25]. Their conclusion was that LTLI correlated strongly with socio‐economic variables.

Bartley and Plewis (2002) examined the accumulation of both disadvantaged class and unemployment over a 20‐year period (1971–1991) from the ONS longitudinal study [England and Wales] to show that belonging to the semi‐ or unskilled social class and being unemployed in 1971 were both related to LTLI in 1991 independently of each other, and of subsequent social class and unemployment [20]. Any further occurrence of disadvantaged social class or of unemployment added significantly to the risk of LTLI.

### 2.2 Geographical Variation in the Prevalence of LTLI

Using data from the 1991 census, Bentham et al. (1995) selected 410 local authority districts in England and Wales and compared the distribution of LTLI between these districts with that of mortality rates and deprivation [26]. The geographical pattern of LTLI showed many similarities with that of mortality and both were positively associated with indicators of social deprivation.

Shouls et al. (1996a and 1996b) examined geographic variation across 278 districts in Britain from the 1991 census of population and uncovered a significant impact of socio‐economic conditions and of urban/rural contrast on LTLI [27, 28].Their results also suggested generally higher levels of ill health for individuals who were older, not married, in a semi/unskilled manual social class, and, as measured by a composite deprivation score, socioeconomically deprived.

### 2.3 Link Between LTLI and Other Illnesses

Cohen et. al. (1995) examined the association between self‐reported LTLI and other dimensions of self‐reported health in Lothian Scotland in 1993 [*n* = 6212] and found that LTLI was strongly associated with physical limitations on activity and less strongly influenced by scores on mental and social well‐being [29]. The socio‐economic effects on LTLI were largely mediated through measures of general health and physical limitations on health. Payne and Saul (2000) examined the relationship between self‐reported LTLI and other disease specific symptoms, mortality and use of hospital services in Sheffield [1994–98] and concluded that LTLI was more prevalent with some conditions (such as angina) than with others (such as gastrointestinal disease) [30]. For all groups, both mortality and, to a lesser extent, hospital admission rates were higher for those with LTLI than those without.

Jordan et. al. (2000) observed that needs assessment had become one of the cornerstones for commissioning health care in the UK and, in the absence of a methodological framework for such assessment, argued for using LTLI as a metric for needs assessment covering a wide range of illnesses [31]. Using data from North Staffordshire, they found LTLI was a good indicator of the general health of the population and therefore a good basis for commissioning services.

### 2.4 Impact on Individuals Living With LTLI

In contrast to the quantitative studies, cited above, McClean (2001) conducted a qualitative study amongst staff with direct work experience in 11 UK social services departments (*n* = 2031 employees) to understand their experiences and found that having a LTLI did not necessarily have a deleterious effect on work skills; if employers were flexible and empathetic in their responses, they could avoid higher sickness levels amongst employees [32].

At the individual level, living with a LTLI imposes financial costs. Blank and Burstrom (2002) looked at the experience of financial strain for those with a LTLI in Sweden using data from the Swedish Survey of Living Conditions [*n* = 41,497] [33]. They found that young people or those living in poor households or persons who were unemployed or early retired had a greater probability of being financially strained by LTLI and might aggravate inequalities between socio‐economic groups.

Those with LTLI also had more acute care needs. Orford et al., (2016) looked at the care needs of LTLI patients referred to intensive care unit by the medical emergency team or direct referral in an Australian tertiary teaching hospital [*n* = 1024] [34]. LTLI patients had a prolonged hospital stay and a high 1‐year mortality with only one‐quarter having documented goals on care. Patients with cancer‐related and frailty‐related LTLI had the worst survival trajectories.

In summary, existing scholarship reveals a clear association between socio‐economic deprivation and the incidence of LTLI, confirmed by the geographical distribution which shows higher patterns in areas of disadvantage. There is also evidence of the link between those with LTLI and the incidence of other illnesses. Those suffering from LTLI were at higher financial risk and imposed greater costs on the health care system.

## 3 Data and Methodology for Analysing Long‐Term Limiting Illness

The UK‐LFS asked its respondents whether they had a long‐term illness; if so, did it limit their activity; and, if it did, whether it limited it a little or a lot. Of these respondents, this paper analysed those who were of working age (16–64 years): 51,814 in 2019 and 36,918 in 2022. Grossing up the sample values for the working‐age respondents by the survey weights provided in the LFS (the variables PWT18 and PWT22 in, respectively, the 2019 and 2022 samples), Table 1 shows that of the total respondents, 67.8% in 2019 and 63.9% in 2022 did not have a long‐term illness (hereafter, N‐LTI), 12.7% in 2019, compared to 13.1% in 2022, had a long‐term illness which was not limiting (NL‐LTI); 11.4% in 2019, compared to 13.3% in 2022, had a long‐term illness which limited a little (hereafter, LTLI‐little); and 8.2% in 2019, compared to 9.7% in 2022, had a long‐term illness which limited a lot (hereafter, LTLI‐lot).

**TABLE 1:** Long‐term illness in the UK's working‐age population, 2019 and 2022 (October–December) (Respondents to UK‐LFS who were of working age (16–64 years): 51,814 and 36,918 respondents in, respectively, 2019 and 2022).

	Percentages of persons who:							
	Do not have a long‐term illness		Have a long‐term illness but it is not limiting		Have a long‐term illness and it limits a little		Have a long‐term illness and it limits a lot	
	2019	2022	2019	2022	2019	2022	2019	2022
All persons	67.8	63.9	12.7	13.1	11.4	13.3	8.2	9.7
Gender								
Male	69.9	67.0	13.3	13.6	9.6	11.0	7.2	8.4
Female	65.7	60.7	12.0	12.7	13.2	15.5	9.2	11.1
Age band								
16–24	76.4	72.1	8.3	8.8	9.4	11.7	5.9	7.3
25–34	76.2	69.2	8.8	10.3	10.0	13.4	4.9	7.1
35–44	73.2	68.9	10.8	11.8	10.0	12.0	6.0	7.4
45–54	62.7	60.1	14.8	15.6	12.5	13.2	10.0	11.1
55–64	51.3	50.3	20.1	18.4	14.8	15.8	13.9	15.5
Country								
England	68.0	64.2	12.7	13.4	11.5	13.3	7.8	9.2
Wales	65.4	58.8	12.8	13.1	11.3	14.3	10.5	13.7
Scotland	65.2	61.1	14.0	12.8	11.5	14.5	9.4	11.7
Northern Ireland	71.5	68.9	8.0	7.8	8.0	8.9	12.5	14.5
Occupational class								
Management/Professional	73.9	71.7	14.4	14.8	9.4	11.1	2.4	2.5
Intermediate	70.7	67.3	13.9	14.2	12.0	14.1	3.4	4.5
Routine non‐manual	70.2	66.4	12.6	12.5	12.2	15.8	5.0	5.4
Routine manual	72.0	67.4	12.5	14.6	11.4	13.5	4.2	4.4
Ethnicity								
White	66.5	62.1	13.3	13.9	11.8	13.8	8.4	10.2
Black	76.2	76.4	8.3	7.0	8.5	8.8	7.0	7.8
Asian	76.7	73.6	9.0	9.4	8.0	10.0	6.4	7.0
Mixed/Other	70.0	67.8	8.9	10.6	12.9	13.5	8.3	8.1
Highest education								
Degree or equivalent	73.7	70.1	13.3	13.9	9.3	11.8	3.7	4.3
Below degree	67.7	63.6	13.5	14.0	12.1	13.6	6.7	8.8
Low	62.5	57.3	11.4	11.6	12.7	14.7	13.4	16.5
Family structure								
Married or co‐habiting	70.9	68.0	13.3	13.6	10.3	11.8	5.8	4.3
Single male	61.5	56.3	12.1	13.9	11.9	13.8	14.4	16.1
Single female	52.6	49.4	12.5	11.3	17.7	20.1	17.2	19.2
Single parent, dependent children	64.0	56.6	9.5	9.6	15.3	17.5	11.2	16.3
Single parent, non‐dependent children	59.0	51.7	12.6	12.5	12.4	16.4	16.0	19.4
Housing tenure								
Owner‐occupier	70.1	68.2	14.2	14.6	10.5	11.6	5.2	6.0
Renting	63.5	56.3	9.9	10.6	13.1	16.2	13.5	17.0

*Note:* Respondents to UK‐LFS who were of Working Age (16–64): 51,814 and 36,918 respondents in, respectively, 2019 and 2022.

*Source:* UK Quarterly Labour Force Survey, 2019 and 2022 (October–December).

The rates of LTLI‐little and LTLI‐lot varied, as expected, by age. As Table 1 shows, in 2019 and 2022, 51.3 and 50.3% of those in the 55–64 age bracket years had N‐LTI (i.e., did not have a long‐term illness) while the corresponding figures for those in the 45–54 age bracket were, respectively, 62.7 and 60.1% for 2019 and 2022. Similarly, in 2019 and 2022, 13.9 and 15.5% of those in the 55–64 age bracket years had a LTLI‐lot while the corresponding figures for those in the 45–54 age bracket were, respectively, 10.0 and 11.1 for 2019 and 2022.

In both 2019 and 2022, women reported lower rates of N‐LTI than men (65.7 vs. 69.9% in 2019 and 60.7 vs. 67% in 2022) but higher rates of LTLI‐little and LTLI‐lot: in 2019, 13.2 (women) versus 9.6 (men) percent for LTLI‐little and 9.2 (women) versus 7.2 (men) percent for LTLI‐lot; in 2022, 15.5 (women) versus 11.0 (men) percent for LTLI‐little and 11.1 (women) versus 8.4 (men) percent for LTLI‐lot.

In 2019 and in 2022, compared to persons in Management/Professional classes, respondents in the routine manual and the routine non‐manual occupational classes reported lower rates of N‐LTI (70.2 (non‐manual) and 72.0 (manual) versus 73.9% in 2019 and 66.4 (non‐manual) and 67.4 (manual) percent versus 71.7% (Management/Professional) in 2022) and higher rates of LTLI‐little and LTLI‐lot: in 2019, 12.2 (non‐manual) and 11.4 (manual) percent versus 9.4% (Managerial/Professional) percent for LTLI‐little and 5.0 (non‐manual) and 4.2 (manual) percent versus 2.4% (Management/Professional) for LTLI‐lot; in 2022, 15.8 (non‐manual) and 13.5 (manual) percent versus 11.1% (Management/Professional) percent for LTLI‐little and 5.4 (non‐manual) and 4.4 (manual) percent versus 2.5% (Management/Professional) for LTLI‐lot.

In terms of ethnicity, compared to Black and Asian respondents, in 2019 and in 2022, White persons reported lower rates of N‐LTI (66.5 vs. 76.2% for Blacks and 76.7% for Asians in 2019 and 62.1 vs. 76.4% for Blacks and 73.6% for Asians in 2022) but higher rates of LTLI‐little and LTLI‐lot than either Blacks or Asians: in 2019, 11.8 (Whites) versus 8.5 (Blacks) and 8.0 (Asians) percent for LTLI‐little and 8.4 (Whites) versus 7.0 (Blacks) and 6.4 (Asians) percent for LTLI‐lot; in 2022, 13.8 (Whites) versus 8.8 (Blacks) and 10.0 (Asians) percent for LTLI‐little and 10.2 (Whites) versus 7.8 (Blacks) and 7.0 (Asians) percent for LTLI‐lot.

In 2019 and in 2022, the *highest* rate of N‐LTI but also the *highest* rate of LTLI‐lot was in Northern Ireland (respectively, 71.5 and 12.5% in 2019 and 68.9 and 14.5% in 2022); compared to Northern Ireland, England had *lower* rate of N‐LTI (respectively, 68.0 and 64.2% in 2019 and 2022) but, among the UK's countries, it also had the *lowest* rates of LTLI‐lot (respectively, 7.8 and 9.2% in 2019 and 2022).

Table 1 also showed that persons whose education level was high (degree level or equivalent) had *higher* rates of N‐LTI than those with either an intermediate (below degree), or a low, level of education (73.7% for high vs. 67.7% for intermediate, and 62.5% for low, education in 2019 and 70.1 for high vs. 63.6% for intermediate, and 57.3% for low, education in 2022). At the other extreme, persons whose education level was high (degree level or equivalent) had *lower* rates of LTLI‐little and LTLI‐lot than those with either an intermediate, or a low, level of education: for LTLI‐lot, 3.7% for high versus 6.7% for intermediate, and 13.4% for low, education in 2019 and 4.3% for high versus 8.8% for intermediate, and 16.5% for low, education in 2022.

In terms of family structure, Table 1 shows that persons who were married or cohabiting had *higher* rates of N‐LTI compared to single men and women: 70.9% for married versus 61.5% for single men, and 52.6% for single women, in 2019 and 68.0 for married versus 56.3% for single men, and 49.4% for single women in 2022. At the other extreme, persons who were married or cohabiting had *lower* rates of LTLI‐little and LTLI‐lot than single men and women: for LTLI‐lot, 5.8% for married versus 14.4% for single men, and 17.2% for single women, in 2019 and 4.3 for married versus 16.1% for single men, and 19.2% for single women in 2022.

Long‐term illness (whether limiting or not it was limiting) was also related to housing tenure. The rate of N‐LTI was considerably *higher* for owner‐occupiers than for tenants (70.1% vs. 63.5% in 2019 and 68.2% vs. 56.3% in 2022) while owner‐occupiers also had a lower rate of LTLI‐little and LTLI‐lot than tenants: for LTLI‐lot, respectively, 5.2 and 13.5% in 2019 and, respectively, 6.0 and 17.0% in 2022.

### 3.1 Multinomial Logit Estimates

The paper estimated a multinomial logit (MNL) model in which, for each respondent *i* (*i = 1*,…,*N*) the dependent variable, *Z*_*i*_ took one of four values: *Z*_*i*_ *= 1*, if respondent *i did not* have a long‐term limiting illness; *Z*_*i*_ *= 2*, respondent *i did* have a long‐term limiting illness, but it *did not* limit their activity; *Z*_*i*_ *= 3*, if respondent *i did* have a long‐term limiting illness, but it *limited their activity a little* (LTLI‐little); *Z*_*i*_ *= 4*, if the respondent *i* did have a long‐term limiting illness and *it limited their activity a lot* (LTLI‐lot).

With *J = 4* mutually exclusive and collectively exhaustive outcomes, indexed *1…J*, where the first outcome (*j = 1)* is taken as the base outcome, the multinomial logit model is defined by a pair of equations. The first, defines the *log odds ratio* of a person *i* being in status *j > 1*, relative to being in the ‘base’ status *j = 1*, as a linear function of $X_{i}=\left\{X_{iK},K=1\text{⋯}K\right\}$, the vector of values of *K* explanatory variables ($X_{i1}=1$) for the person.

(1) $$\log\left(\frac{\mathrm{Pr}\left(Y_{i}=j\right)}{\mathrm{Pr}\left(Y_{i}=1\right)}\right)=\sum_{k=1}^{K}\beta_{jk}X_{ik}=X_{i}\beta_{j}$$

where *Y*_*i*_ is an integer variable which takes the value *j* if, and only if, outcome *j* occurs for person *i*, and $\beta_{j}$ is the vector of coefficients associated with outcome *j*, $\beta_{j1}$ being the coefficient associated with the intercept term. The second equation defines the probability of outcome *j* (*j = 1…J*) occurring for individual *i* as:

(2) $$\mathrm{Pr}\left(Y_{i}=j\right)=\exp\left(Z_{ij}\right)/\left[1+\sum_{r=1}^{J}Z_{ir}\right]=F\left(X_{i}\beta_{j}\right)$$

where: $\sum_{j=1}^{4}\mathrm{Pr}\left(Y_{i}=j\right)=1$

The MNL model, encapsulated in Equations (1) and (2) was estimated, separately, on data for 2019 (39,410 observations) and 2022 (27,852 observations). The determining variables used in the estimation were as shown in Table 1. Using the estimates of $\beta_{j}$, obtained from estimating Equation (1), the probabilities of every individual being in each of the health categories *(j = 1*,…,*4)* could be estimated using Equation (2).

For both years the estimation was conducted using the LFS‐provided survey weights to gross up the sample. This was done by using the *svy* command in the econometric package Stata: this fits statistical models for complex survey data by adjusting the results of a command for survey settings identified by *svyset*. The survey settings were the person's number (PERSNO) and the weight attached (PWT22 for the 2022 Survey and PWT18 for the 2019 Survey).

### 3.2 Predicted Probabilities

The results are presented in terms of *predicted probabilities*. These are described in Long and Freese (2014, chapter 4) and in a Stata manual and are based on the method of ‘recycled predictions’ [35].

Suppose that one is interested in identifying the probability of persons belonging to the different occupational classes having a LTLI‐lot (or LTLI‐little) which can be *entirely* ascribed to their occupational class. Note that simply computing the probabilities of LTLI‐lot (using Equation (2)) over the four occupational class subsamples (Management/Professional; Intermediate; Routine non‐Manual; Routine Manual) would not provide this answer because, for example, the Managerial/Professional and the Routine Manual subsamples would differ in more than just the occupational class of the individuals. These subsamples might differ in terms of gender, age, country, ethnicity, education, housing tenure and so one could not attribute the subsample difference in probabilities of having a LTLI‐lot *solely* to the ‘occupational class’ effect.

The effect of gender on the PP of LTLI (the average propensity to LTLI) would be contingent on the year in question: 2019 (pre‐Covid) or 2022 (post‐Covid). The average PP of LTLI for men, or for women, could alter significantly between the pre‐ and post‐Covid years, 2019 and 2022. Within the context of this ‘interaction’ model, it was possible to test whether the *difference* between 2019 and 2022, in the average PP of LTLI for a particular group of persons (say, men or women) was significantly different from zero.

To isolate the class effect first treat *all* the individuals in the data as belonging to the Managerial/Professional class and then apply the values of the observed characteristics (gender, age, country, ethnicity, education, housing tenure) of the individuals in the sample to calculate the probability, using Equation (2), of having a LTLI‐lot. Next, treat *all* the individuals in the data as belonging to the Routine Manual and then apply the values of the observed characteristics of the individuals in the sample to calculate the probability, using Equation (2), of having a LTLI‐lot. The difference between the two sets of calculated probabilities of having a LTLI‐lot is *entirely* the result of being from the Managerial/Professional or the Routine Manual classes because, in both calculations, the values of the characteristics (gender, age, country, ethnicity, education, housing tenure) used to compute these probabilities were identical. The probabilities calculated using this methodology are, hereafter, referred to as predicted probabilities (PP).

## 4 Estimation Results From the Multinomial Logit Model

Table 2 shows the results from estimating Equation (1), separately for 2019 and 2022, on UK‐LFS data *for working‐age respondents*. The column labelled ‘Predicted Probability’ shows the PP, as defined above, of the various categories of variables for two aspects of long‐term illness: *no long‐term illness* (N‐LTI) and *long‐term illness which limits a lot* (LTLI‐lot). So, in terms of occupational classes, Table 2 shows that the PP of those in the Managerial/Professional class having N‐LTI and LTLI‐lot in 2019 was, respectively, 73.4 and 2.6% while, in 2022, these PP were 71.0 and 2.8%. In contrast, the PP of those in the Routine non‐Manual class having NLTI and LTLI‐lot in 2019 was, respectively, 69.4 and 4.8% while, in 2022, these PP were 66.3 and 5.0%. As discussed above, these represent the ‘pure’ class effect of having N‐LTI and LTLI‐lot because they were computed with the values of the non‐class variables unchanged. These non‐class variables were, as shown in Table 2: gender; age; country; ethnicity; highest education; family structure; and housing tenure.

**TABLE 2:** Predicted probabilities of long‐term illness in the United Kingdom, 2019 and 2022 (October–December).

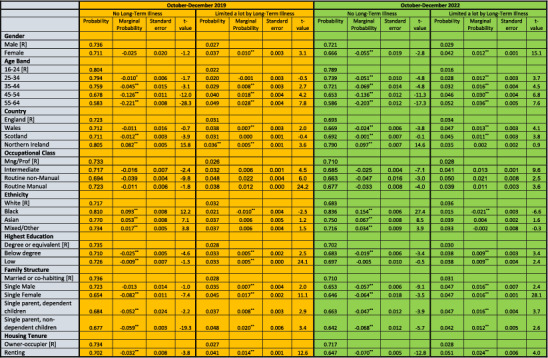

*Note:* Multinomial Logit estimates based on 39,410 and 27,852 observations for, respectively, 2019 and 2022. [R] denotes reference category.

^a^ Marginal probability significant at 5% level.

^b^ Marginal probability significant at 10% level.

*Source:* Own calculations from UK Quarterly Labour Force Survey, 2019 and 2022 (October‐December).

The PP shown in Table 2 should be interpreted as the *average propensity* of each group for LTLI in terms of either its absence (N‐LTLI), or in terms of its intensity (LTLI‐lot). The difference in PP between a group's categories measures the *marginal propensity* (or gradient) of LTLI with respect to that group. In terms of occupational class, the difference between the PP for different occupational classes measures the occupational class gradient of LTLI. These gradients are measured in terms of ‘marginal probabilities’ and discussed below.

The column in Table 2 labelled ‘Marginal Probability’ represents, the average difference in PP between those in a particular category of a variable and those in the reference category, denoted by [R], for that variable. So, for the occupational class category, the numbers in the ‘Marginal Probability’ column represent *differences* between the PP of the individuals in the Intermediate and the Routine Manual and the Routine non‐Manual classes and those in the reference (Managerial/Professional) class, denoted by [R]. The ratios of these marginal probabilities and their standard errors (shown in the next column) are the t‐values. These tested the null hypothesis that the marginal probabilities were zero.

A marginal effect relates to the change in the value of a dependent variable consequent upon a small change in an independent variable, if the latter is a continuous variable, and, if the latter is a discrete variable, a change from one category to another of the independent variable. Since, in the empirical work, the equation estimated is probabilistic, and the independent variables used are discrete, the marginal effect relates to the change in the *probability of the event occurring* following a category change in the relevant independent variable—say from Management/Professional to Routine Manual if the independent variable refers to occupational class. By virtue of the construction of the logistical probability distribution, which underpins the MNL model, the probabilities are constrained to take values between (and including) 0 and 1.

The main results that emerged from the estimation results shown in Table 2 were:

First, in terms of gender, in 2019 there was no significant difference between women and men in the PP of N‐LTI (73.6 vs. 71.1% in 2019 with marginal probability of −0.025) but the PP of having a LTLI‐lot was significantly higher for women than for men (3.7 vs. 2.7% with marginal probability of 0.010). In 2022, however, the PP of N‐LTI was significantly lower for women than for men (66.6 vs. 72.1% with a marginal probability of −0.055) and the PP of LTLI‐lot was significantly higher (4.2 vs. 2.9% with a marginal probability of 0.012).

Second, in terms of occupational class, persons in the Managerial/Professional classes had a significantly *higher* PP of N‐LTI (i.e., of not having a long‐term illness) than persons either in the Routine non‐Manual or Routine Manual classes and also had a significantly *lower* PP of LTLI‐lot (i.e., of having a long‐term illness which limited activity by a lot) than persons either in the Intermediate or in the Routine Manual/non‐Manual classes. This was true in both 2019 and 2022.

We also tested the null hypothesis that the PP of N‐LTI and LTLI‐lot was the same for persons in the Routine Manual and Routine non‐Manual classes. For 2019, the PP of N‐LTLI for persons in the Routine Manual classes (72.3%) was *significantly higher*, and PP of LTLI‐lot *significantly lower*, than that for those in the Routine non‐Manual classes (N‐LTLI: 72.3 vs. 69.4%; LTLI‐lot: 3.8 vs. 4.8%). However, in 2022, there was no significant difference between the two classes in their respective PP of N‐LTLI or in their respective PP of LTLI‐lot.

Lying outside the scope of this paper is the impact that LTLI might have on the differential employment prospects of persons in manual and non‐manual occupations. People with better employment conditions might be able to continue working, while those working under less favourable conditions might have to give up their jobs, even though both sets of persons had the same degree of illness. The implication of this is that job loss and economic activity, related to ill‐health, followed a social gradient which mirrored the social gradient to ill‐health (Minton et. al. 2012) [36].

Third, in terms of education, persons who had a degree (or equivalent), in 2019 and in 2022, had a significantly *higher* PP of N‐LTI (respectively, 73.5 and 70.2%) than persons with a below degree level of education (respectively, 71.0 and 68.3%) but, in *neither* year was there a significant difference in the PP of N‐LTI between persons who had a degree (or equivalent) and those who had a low level of education in (72.6 and 69.7% in 2019 and 2022, respectively, for those with low education). In both 2019 and 2022, however, persons who had a degree (or equivalent) had a significantly lower PP of having LTLI‐lot than persons with a below degree level of education (2.8 vs. 3.3% for 2019 and 3.0 vs. 3.8 for 2022) and persons with a low level of education (2.8 vs. 3.3% for 2019 and 3.0 vs. 3.8 for 2022).

Comparing persons with a below degree education level with those whose education level was low, the PP of having N‐LTI in 2019 and in 2022 was significantly lower for the ‘below degree’ than for the ‘low education’ group (71.0 vs. 72.6% in 2019 and 68.3 vs. 69.7% in 2022). In neither year, however, was there a significant difference between the two groups in the PP of LTLI‐lot.

Fourth, in terms of ethnicity, in both 2019 and 2022, White persons classes had a significantly *lower* PP of N‐LTI than Black or Asian persons (71.7 vs. 71.0 (Black) and 77.0 (Asian) percent in 2019 and 68.3 versus 83.6 (Black) and 75.0 (Asian) percent in 2022); White persons also had a significantly *higher* PP of LTLI‐lot than Black persons (3.2 vs. 2.1% in 2019 and 3.6 vs. 1.5% in 2022) but there was no significant difference between Whites and Asians in the PP of having a LTLI‐lot (3.2 vs. 3.7% in 2019 and 3.6 vs. 3.9% in 2022).

Comparing Blacks with Asians showed that the PP of N‐LTI of Blacks was significantly *higher* than that of Asians in 2019 and 2022 (respectively, 81.0 and 77.0% in 2019 and 83.6 and 75.0% in 2022) the PP of LTLI‐lot of Blacks was significantly *lower* than that of Asians in 2019 and 2022 (respectively, 2.1 and 3.7% in 2019 and 1.5 and 3.9% in 2022).

Fifth, in terms of family type, in 2019 and 2022, persons who were married or cohabiting had a significantly *higher* PP of N‐LTI than single females (respectively, 73.6% and 65.4% in 2019 and 71.0 and 64.6% in 2022), and a significantly *lower* PP of LTLI‐lot (respectively, 2.8 and 4.5% in 2019 and 3.1 and 4.7% in 2022). This was also true of persons who were married or cohabiting vis‐à‐vis single males in 2022 in respect of N‐LTI (respectively, 71.0 vs. 65.3%) and LTLI‐lot (respectively, 3.1 vs. 4.7%) but, in 2019, there was no significant difference between married or cohabiting persons and single males in their PP of N‐LTI.

In consequence, while in 2019, single males had a significantly *higher* PP of N‐LTI, and a significantly *lower* PP of LTLI‐lot, than single females, by 2022 these differences had disappeared—there was, in 2022, no significant difference between single males and females in their PP of N‐LTI and LTLI‐lot.

Lastly, in terms of housing tenure, in both 2019 and 2022, persons who were owner‐occupiers had a significantly *higher* PP of N‐LTI than those who rented (73.4% vs. 70.2% in 2019 and 71.7 vs. 64.7% in 2022) and had a significantly *lower* PP of a LTLI‐lot (2.7 vs. 4.1% in 2019 and 2.8 vs. 5.1% in 2022).

## 5 Pre‐ and Post‐Covid Comparison of Health Outcomes

The previous sections discussed results for long‐term health outcomes for the pre‐ and post‐Covid years, 2019 and 2022, when each year was considered in isolation. This section turns to a comparison of LTLI outcomes between the two years in the context of a model in which the ‘LTLI equation’ (detailed in Table 2) is estimated on data *pooled* over 2019 and 2022. By comparing the year just before the Covid pandemic (2019) with that just after (2022), we can examine whether the propensity to LTLI was significantly altered by the pandemic? Or, in other words, was there a significant difference between the pre‐ and post‐Covid periods in the average propensity to LTLI both in aggregate and in respect of the individual covariates?

Within this pooled dataset, the variable *C* was used to define the year of the Survey: for *N* respondents, indexed *i = 1…N*, *C*_*i*_ *= 1* if the data for respondent *i* was for 2019 and *C*_*i*_ *= 2* if the data for respondent *i* referred to 2022.

Following this, every component of the vector of determining variables, **x**, in the health equation, was allowed to interact, with the ‘year’ variable, *C*:

(3) $$Y_{i}=f\left(x\times C_{i}\right)$$

Table 3 shows the results of comparing the average PP of LTLI between 2019 and 2022. The left‐hand panel shows the PP of not having a long‐term illness (N‐LTI) and the panel on the right shows the PP of having a long‐term illness which limits a lot (LTLI‐lot), both sets of PP computed over the 67,262 respondents in the pooled sample of which 39,410 and 27,852 were from 2019 to 2022, respectively. The second and third columns of Table 3 show the PP associated with, respectively, 2019 and 2022; the fourth column computes the difference between these two sets of PP; the fifth column shows the standard associated with this difference; and the sixth column computes the z‐value as the ratio of the difference and the standard error with an asterisk(s) indicating whether the difference was significantly different from zero. As discussed earlier, these predicted probabilities were computed by assuming that *all* the 67,262 respondents were first from the pre‐Covid year (2019), and then from the post‐Covid year (2022), the values of the other variables remaining unchanged at their observed sample values between these two scenarios.

**TABLE 3:** Differences between 2019 and 2022 in the predicted probabilities of long‐term illness in the United Kingdom.

	No long‐term illness					Limited a lot by long‐term illness				z‐value
	Probability 2019	Probability 2022	Difference	Standard error	z‐value	Probability 2019	Probability 2022	Difference	Standard error	
Overall	0.724	0.694	0.031^a^	0.006	5.4	0.031	0.035	−0.004^b^	0.002	−1.8
Gender										
Male	0.737	0.720	0.016^a^	0.006	2.6	0.026	0.029	−0.003	0.004	−0.8
Female	0.712	0.665	0.046^a^	0.005	8.6	0.037	0.042	−0.005^a^	0.001	−3.5
Age band										
16–24	0.805	0.789	0.016	0.010	1.6	0.021	0.016	0.005^a^	0.002	2.6
25–34	0.795	0.738	0.057^a^	0.012	4.9	0.020	0.028	−0.008^a^	0.003	−2.6
35–44	0.760	0.720	0.040^a^	0.003	15.8	0.029	0.032	−0.003	0.003	−1.0
45–54	0.679	0.652	0.027^a^	0.001	25.7	0.040	0.046	−0.007^a^	0.002	−3.9
55–64	0.584	0.585	0.000	0.012	0.0	0.049	0.053	−0.004^b^	0.002	−1.7
Country										
England	0.724	0.692	0.032^a^	0.007	4.6	0.031	0.034	−0.003	0.002	−1.3
Wales	0.713	0.668	0.045^a^	0.013	3.4	0.038	0.047	−0.009^b^	0.005	−1.7
Scotland	0.712	0.691	0.021^a^	0.007	3.1	0.031	0.045	−0.014^a^	0.004	−3.2
Northern Ireland	0.806	0.790	0.016^a^	0.005	3.0	0.036	0.036	0.000	0.002	0.1
Occupational class										
Management/Professional	0.734	0.710	0.024^a^	0.005	5.1	0.026	0.028	−0.002	0.003	−0.7
Intermediate	0.718	0.684	0.033^a^	0.013	2.6	0.032	0.041	−0.009^a^	0.003	−3.0
Routine non‐manual	0.695	0.663	0.032^a^	0.011	3.0	0.048	0.050	−0.002	0.010	−0.2
Routine manual	0.723	0.677	0.047^a^	0.007	6.2	0.038	0.039	−0.001	0.000	NA
Ethnicity										
White	0.717	0.682	0.035^a^	0.006	5.7	0.031	0.036	−0.005^a^	0.002	−2.0
Black	0.810	0.836	−0.026^a^	0.011	−2.4	0.021	0.015	0.006	0.005	1.3
Asian	0.770	0.749	0.021^a^	0.007	3.1	0.037	0.040	−0.003	0.005	−0.5
Mixed/Other	0.735	0.716	0.018	0.015	1.2	0.037	0.034	0.003	0.006	0.5
Highest education										
Degree or equivalent	0.735	0.701	0.033^a^	0.008	4.3	0.028	0.030	−0.002	0.001	−1.3
Below degree	0.710	0.682	0.028^a^	0.004	6.9	0.033	0.038	−0.005^a^	0.002	−3.0
Low	0.726	0.696	0.030^a^	0.007	4.2	0.033	0.038	−0.005	0.005	−1.1
Family structure										
Married or co‐habiting	0.737	0.709	0.028^a^	0.007	3.9	0.028	0.031	−0.003	0.003	−0.9
Single male	0.724	0.652	0.072^a^	0.012	6.1	0.035	0.048	−0.013^b^	0.007	−1.8
Single female	0.655	0.645	0.011^a^	0.004	2.6	0.045	0.047	−0.003	0.003	−0.8
Single parent, dependent children	0.685	0.662	0.023	0.026	0.9	0.036	0.047	−0.011^b^	0.006	−1.8
Single parent, non‐dependent children	0.678	0.641	0.037^a^	0.007	5.6	0.048	0.043	0.005	0.004	1.3
Housing tenure										
Owner‐occupier	0.735	0.716	0.019^a^	0.007	2.8	0.027	0.028	−0.001	0.001	−0.6
Renting	0.703	0.646	0.057^a^	0.004	15.5	0.041	0.052	−0.011^b^	0.006	−1.8

*Note:* Multinomial Logit estimates based on 67,262 pooled observations: 39,410 and 27,852 for, respectively, 2019 and 2022.

^a^ Difference significant at 5% level.

^b^ Difference significant at 10% level.

*Source:* Own calculations from UK Quarterly Labour Force Survey, 2019 and 2022 (October‐December).

The main results to emerge from this analysis can be summarised as follows:

First, for the overall sample, the difference in the PP of N‐LTI (respectively, 72.4 and 69.4% for 2019 and 2022) and the PP of LTLI‐lot (3.1 and 3.5% for 2019 and 2022) were both significantly different from zero, though the latter difference was only significant at the 1% level.

Second, both men and women had a significantly higher PP of N‐LTI in 2019 than in 2022; furthermore, women, though not men, also had a significantly higher probability of LTLI‐lot in 2022 than in 2019.

Third, in every country, the PP of N‐LTI was significantly higher in 2019 than it was in 2022 but it was only in Scotland that there was a significant difference between the two years in LTLI‐lot with the 2022 LTLI‐lot PP for Scotland being significantly higher in 2022 than it was in 2019.

Fourth, for each of the four occupational classes, the PP of N‐LTI was significantly higher in 2019 than in 2022, but it was only for the Intermediate occupational class that the PP for LTLI‐lot in 2022 was significantly higher than the corresponding 2019 values (Intermediate: 3.2 to 4.1%).

Fifth, in terms of ethnicity, the PP of N‐LTLI *fell* significantly for White and Asian respondents between 2019 and 2022 (Whites: 71.7 to 68.2%; Asians: 77 to 74.9%); fell, but not significantly, for Mixed Race respondents (73.5–71.6%); and *rose* significantly for Black respondents (from 81 to 83.6%). The PP of LTLI‐lot rose significantly between 2019 and 2022 for White respondents but was statistically unchanged for the other ethnicities.

Sixth, in terms of the highest level of education, the PP of N‐LTI was significantly higher in 2019 than in 2022 for each of three education levels (Degree: 73.5 to 70.1%; Below Degree: 71 to 68.2%; Low: 72.6 to 69.6%) but it was only for the Below Degree respondents that the PP for LTLI‐lot in 2022 was significantly higher than the corresponding 2019 values (3.3–3.8%).

Seventh, or the five types of family structure, except for single parents with depend children, the PP of N‐LTI was significantly higher in 2019 than in 2022 (Married: 73.7 to 70.9%; Single Male: 72.4 to 65.2%; Single Female: 65.5 to 64.5%; Single Parent with dependent children: 68.5 to 66.2%; Single Parent with non‐dependent children: 67.8 to 64.1%) but it was only for single males and single parents with dependent children that the PP for LTLI‐lot in 2022 was significantly higher (and that only at a 10% significance level) than the corresponding 2019 values.

Eighth, for both types of housing tenure, the PP of N‐LTI was significantly higher in 2019 than in 2022 (Owner‐Occupiers: 73.5 to 71.6%; Renting: 70.3 to 64.6%), but it was only for those renting their accommodation that the PP for LTLI‐lot in 2022 was significantly higher (and that only at a 10% significance level) than the corresponding 2019 values.

The overall result to emerge from this section's analysis is that the average PP of not having a long‐term illness (N‐LTI), for most groups, *fell* significantly between 2019 and 2022; to put it differently, the PP of having a long‐term illness ‐ whether limiting or not and, if limiting, limiting by a little or a lot ‐ was significantly *higher* in 2022 than it was in 2019. However, there were far fewer instances of persons having a significantly *highe*r PP of being limited *a lot* by long‐term illness (LTLI‐lot) in 2022 than in 2019.

## 6 Discussions

Between August‐October 2024 and January March 2020, the number of economically inactive working‐age persons increased by approximately 700,000: from 8.6 million in 2020 to 9.3 million in 2024. A major factor contributing to this rise has been long‐term limiting illness (Andrews, 2024) [37].

With this background, the purpose of this paper was to study the evolution of LTLI in the UK between the pre‐ and post‐Covid years of, respectively, 2019 and 2022 paying attention to differences in the propensity to LTLI between different subgroups of the population *in* each of the two years and then examining whether the propensity to LTLI changed *between* the years, both in respect of overall change and in respect of the separate population subgroups.

In terms of the social gradient to health, persons in the Managerial/Professional classes had a significantly *higher* PP of N‐LTI (i.e., of not having a long‐term illness) than persons either in the Routine non‐Manual or Routine Manual classes and also had a significantly *lower* PP of LTLI‐lot (i.e., of having a long‐term illness which limited activity by a lot) than persons either in the Intermediate or in the Routine Manual or Routine non‐Manual classes. This was true in both 2019 and 2022. In other words, there was significant inequality in the PP of LTLI associated with the occupational classes.

In terms of changes in the propensity to LTLI, the PP of having a long‐term illness—regardless of whether it was limiting or not ‐ was significantly *higher* in 2022 than it was in 2019 both for the overall population and for its subgroups. This increase in propensity to LTLI affected both genders, all occupational classes, all the countries of the UK, all ethnicities (except Blacks), all levels of education, all family types (except single parents with dependent children), and all housing tenures. However, there were far fewer instances of subgroups having a significantly *highe*r PP of being limited *a lot* by long‐term illness (LTLI‐lot) in 2022 than in 2019.

These results for the UK may be compared with corresponding results for France. Lahbib et. al. (2024) [15], used data from four French Health Barometer Surveys (2017, 2019, 2020, and 2021) to assess the evolution of Self‐Rated Health (SRH) and activity limitation (measured by a Global Activity Limitation Indicator—GALI) of people in France aged 18–75 years. They found that between 2017 and 2021, both SRH and GALI deteriorated in France. This deterioration affected all classes, both genders, and all age‐classes, and all geographical regions. In short, the results for France mirror the results reported here for the UK.

Recent and related studies have examined the impact of long COVID on life limiting illnesses. Work by Carlile et al. (2024) examined the differences in health impacts of long COVID across sociodemographic categories and quantified these using Quality‐Adjusted Life Years. They found substantial impacts on quality of life due to long COVID placing a major burden on patients and the health service [38]. Sandmann et al. (2022) reported on non‐hospitalised COVID cases and the impact on their long‐term health‐related quality of life and found that 10% reported (*n* = 548 cases) cases suffered prolonged loss of function compared to pre‐COVID baselines [39].

## 7 Conclusions

The main findings of this pre‐and post‐COVID study confirm there is significant inequality in the predicted probability of long‐time limiting illness associated with occupational classes. Managerial/Professional classes had a significantly higher predicted probability of not having a long‐term illness both before and after COVID. However, the predicted probability of having a long‐term illness was significantly higher in 2022 than it was in 2019 both for the overall population and for its subgroups. A particular value of this analysis is to provide empirical evidence for the UK that the impact of Covid has been to exacerbate health inequalities. Importantly, the concentration of work on mortality has overshadowed research on morbidity, yet the significance of morbidity to economic performance is critical in terms of persons being, or becoming, economically inactive.

This study also has important policy implications. State support for those living with long‐term limiting illness is complex. Those eligible can claim a Personal Independence Payment (PIP) which is designed to help with some of the extra costs caused by their illness. The benefit is not means tested and comprises two parts: a daily living component and a mobility component. The amount one receives depends on how one is affected by the condition, not the condition itself. To qualify, people must have a health condition or disability where they have had difficulties with daily living or getting around (or both) for 3 months, and where it is expected that these difficulties will continue for at least 9 months.

Those unable to work may be eligible for Universal Credit depending on their circumstances. There is limited evidence on either the consistency of the assessment process for PIP and its impact on ability of those suffering for long‐term limiting illness to continue in employment. Criticisms include the complexity of the application process, delays and backlogs in the assessment of applicants, inaccurate assessments and lack of flexibility in the benefits system to accommodate the changing needs of those with long‐term limiting illness.

Given the increasing prevalence of long‐term limiting illness, this policy area has prompted a radical overhaul in how the state deals with the problem and a review of the benefits system in place to assist those people debilitated with such illnesses. Former Prime Minister Sunak launched a review (April 2024) to tackle this issue claiming that the government spends £69bn on benefits for people of working age with a disability or health condition, more than the schools' budget, transport budget or policing budget. In 2019, there were an average of around 2200 new PIP awards a month in England and Wales where the main condition was anxiety and depression ‐ this has more than doubled to 5300 a month in 2023. This has driven up the cost of the disability benefits bill at an unsustainable rate and PIP spending alone is expected to grow by 52% from 2023/24 to £32.8bn by 2027/28 (Prime Minister's office Press Release, 19^th^ April 2024) [40]. The proposals are part of a wider reform package billed as a new ‘moral mission’ to reform the welfare system which controversially would remove ‘fitness to work’ certificates from GPs to specialist work and health professionals.

Proposed changes to PIP include replacing cash payments with medical treatment to improve health conditions, greater medical evidence to substantiate a claim, and a higher threshold for eligibility. All of this is to address increasing economic inactivity and especially rising inactivity due to long‐term sickness. In its consultation on long‐term limiting illness the government is seeking advice for those ‘with an understanding of the system’. This research paper contributes to the policy narrative by offering empirical evidence on the groups most susceptible to long‐term limiting illness before and after Covid.

More widely there are a number of policy implications arising from this and related research on the impact of long COVID. Healthcare systems will need to consider the budgetary implications of managing long‐term conditions including further investment in disability and rehabilitation services. There will be greater demand for social security and disability benefits across OECD countries (and beyond) which may require adjustments to welfare policies. Higher rates of long‐term life limiting illnesses may reduce workforce participation which could cause labour shortages in specific sectors and lower productivity. These trends will demand increased spending on healthcare costs and social welfare payments at a time when governments are reluctant to increase taxation. As the evidence in this paper demonstrates, long‐term limiting illness has a disproportionate impact on vulnerable groups, hence socio‐economic disparities are likely to widen inequality.

The research also has important limitations. A before and after study cannot capture the potential longer‐term impact of COVID on long term life limiting illnesses given the possibility of a time lag for the progression of serious health conditions. Patients with long‐term conditions often have comorbidities and diverse socio‐economic backgrounds which makes it difficult to draw causal inferences directly to COVID. Long‐term illnesses may be underdiagnosed, particularly in low‐income groups and self‐reported symptoms may be unreliable which makes for gaps in the data.

These limitations offer opportunities for future research—large scale longitudinal and cohort studies; social and economic impact research which assesses the long‐term economic burden of life‐limiting illnesses on healthcare systems, labour markets and social welfare programmes; and medical research (clinical trials and non‐pharmaceutical interventions) to tackle long‐term illnesses.

## Ethics Statement

The authors have nothing to report.

## Conflicts of Interest

The authors declare no conflicts of interest.

## Data Availability

The data used in this paper can be accessed at: https://beta.ukdataservice.ac.uk/datacatalogue/series/series?id=2000026.
